# Estimating hydrogen usage of a Crew Transport Vessel fleet for Offshore Windfarm maintenance

**DOI:** 10.1007/s13437-023-00300-x

**Published:** 2023-02-01

**Authors:** Habbo Cramer, Annika Fitz, Arto Niemi, Bartosz Skobiej, Frank Sill Torres

**Affiliations:** 1 Institute for the Protection of Maritime Infrastructures, German Aerospace Center (DLR), Fischkai 1, Bremerhaven, 27572 Germany; 2 Bremenports GmbH & Co. KG, Am Strom 2, Bremerhaven, 27568 Germany; 3 Institute of Maritime Energy Systems, German Aerospace Center (DLR), Max-Planck-Straße 2, Geesthacht, 21502 Germany

**Keywords:** Hydrogen, Offshore wind farm, Maintenance, Crew transport vessel

## Abstract

This paper presents an estimate for the mass of hydrogen that would be needed to power the current fleet of Crew Transport Vessels (CTVs) used for maintaining the German offshore wind farms and how this demand may be geographically distributed. The estimate is based on a calculation of the marine diesel oil consumption of the current fleet, which is further employed for estimating the current emissions of these activities. Based on the predicted price of hydrogen, bunkering the CTV fleet with hydrogen may become economically advantageous in the future. Moreover, using hydrogen may reduce CO_2_ emissions. Results have been obtained by using vessel position data, weather data, and diesel consumption estimates. As a shortcoming, certain effects are excluded from the current estimate. However, this work presents an approach that can be improved and used for estimating hydrogen consumption in future scenarios. In these scenarios, a vessel type and parameters can be set. While here the challenge was to create a generic model that can be applied to multiple types of vessels.

## Introduction

Decarbonization of the maritime industry is an essential aspect of slowing the global warming that is driven by greenhouse gases. Several governments haven taken action to reduce these emissions. In case of the German government, its hydrogen strategy aims to establish hydrogen as a decarbonization option (BMWK 2020). Its is intended that the push for green energy production in Germany will increase the reliance on offshore wind farms (OWFs) that provide the option for Offshore hydrogen production (Bundesrat 2022). These plans call for the identification of areas that can be used for such production sites (BMWK 2020), while the offshore wind energy law anticipates the construction of hydrogen pipelines (Bundesrat 2022).

However, a common challenge of renewable energy sources is its inconstant production rate, which depends, for example on wind speed or the amount of solar radiation. The so-called “Power to X” concept aims to solve this issue by transforming electricity into another form that permits the storage of energy such that it can be provided when needed (Wulf et al. 2020). For example in Germany, the AquaVentus initiative pursues this concept for OWF, i.e. the production of hydrogen with the energy provided by OWFs in the North Sea (AquaVentus Förderverein 2020). Similar initiatives can be seen in Denmark (Ørsted ), Germany (Fichter 2022) and the United Kingdom (Ørsted ). Therefore, one can assume that hydrogen should be readily available in future OWFs.

Availability of the hydrogen will create an opportunity to use it to power the vessels needed for OWF installation and operational phases. During the operations, Crew Transport Vessels (CTVs) are used for transporting maintenance personnel to perform their activities. As will be described in Section 2, currently these vessels employ fossil Marine Diesel Oil (MDO). Thus, replacing such propulsion systems against hydrogen based solutions one can further reduce the carbon footprint of OWFs.

Our contribution presents a calculation of the amount of hydrogen needed to maintain the current German OWFs and how this demand may be geographically distributed. This calculation is based on OWF maintenance operations, as observed in stored Automatic Information System (AIS) data. We further use this calculation for estimating the current emissions of these activities. Based on the predicted price of hydrogen, bunkering the CTV fleet with hydrogen may become economically advantageous in the future. Moreover, using hydrogen may reduce CO_2_ emissions.

Maritime vessels use the AIS to communicate their locations to other vessels and vessel tracking services. Section 3 provides details on material for calculation and Section 4 on the methods. The results are presented in Section 5.

There are two issues in the presented results. Estimating the effects of the weather on small vessels is complicated due to various hull shapes and the lack of estimates that are available only for large ships (Molland et al. 2011). Secondly, the authors had access to a limited data set for validating results. Section 6 discusses the validation issue and the paper ends with Section 7 on conclusions and future work.

## Background on crew transport vessels and their propulsion

**Fig. 1:**
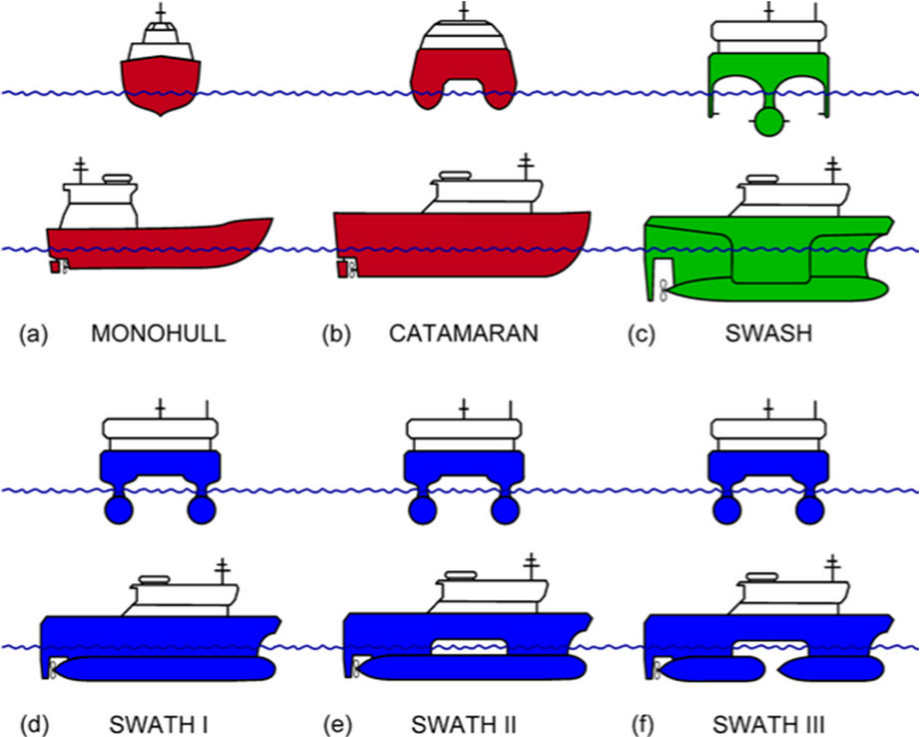
Comparison of hull shapes for coastal vessels (SWASH - Small Waterplane Area Single Hull, SWATH - Small Waterplane Area Twin Hull) (Łebkowski and Koznowski 2020)

**Fig. 2:**
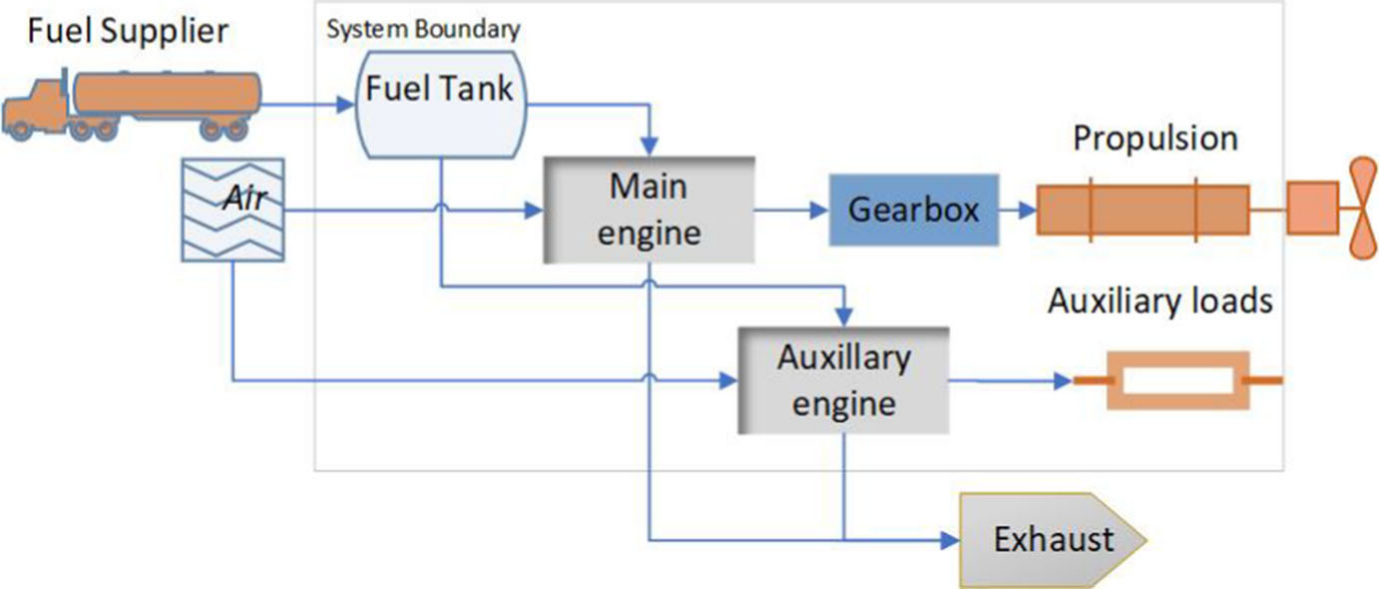
Illustration of a diesel powering system onboard a CTV

A CTV is a ship that is used to transport service technicians between shore and OWF (Almat 2015; Łebkowski 2020). Since a CTV is only designed for transport, the ship is significantly smaller than a service operation vessel. Figure 1 depicts different types of hulls used in coastal vessels^1^. Today, these vessels are most commonly powered by diesel engines. Figure 2 illustrates this powering system.

Several tests have been conducted to use fuel cells to power small vessels or as an auxiliary power unit for large vessels (Markowski and Pielecha 2019). Furthermore, the use of fuel cells in coastal vessels has been studied by (Łebkowski and Koznowski 2020; Łebkowski 2020). However, a review of the potential of hydrogen in maritime applications concluded that hydrogen fuel cells will not replace the existing multi-megawatt main engines of large ships in the foreseeable future (Vogler and Würsig 2011). However, hydrogen-fueled systems are a valid option for low power demands or when only a regional fuel supply is required.

**Fig. 3:**
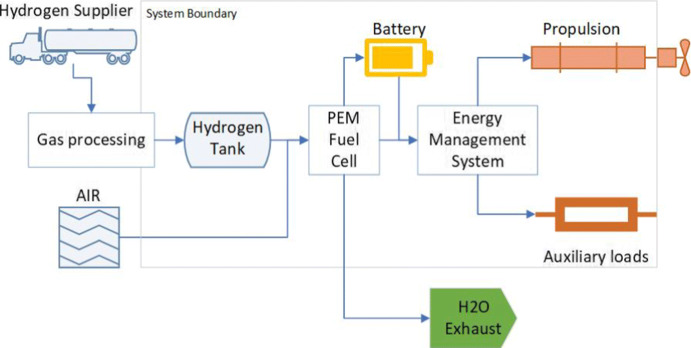
Illustration of a hydrogen fuel cell powering system onboard a CTV

Figure 3 illustrates a hydrogen-powered fuel cell propulsion in a CTV, where hydrogen is fed to a Polymer-Electrolyte Membrane (PEM) fuel cell. Produced electricity can power the vessel or it can be stored in a battery. These types of fuel cells have several advantages: (1) high electricity production efficiency, (2) good response time of cell systems, (3) short start-up time, and (4) low operating temperature (Markowski and Pielecha 2019). Therefore, they have been the most popular option in ship projects.

We believe that other types of fuel cells suited for maritime use like Molten Carbonate Fuel Cells (MCFC) and Solid Oxide Fuel Cells (SOFC) are currently not an option to provide propulsive load for a CTV, due to their lack in transient capacity. Ongoing research aims at integrating them for Hotel load, e.g. on cruise ships. For MCFC, the challenges originate from the mobile and corrosive electrolyte, which corrodes electrode and construction materials and degrades the mechanical stability and lifetime (Jiang and Li 2022). The fuel cell power output is commonly below 200 mW/cm^2^, limiting the technology for stationary applications in a power range from 300 kW to 3 MW. SOFCs have potential for medium to long distance ship applications. However, due to the slow start-up and load shifting, SOFCs are expected to handle base loads of thermal and electric energy demand (Baldi et al. 2020). Consequently, it should be integrated in to hybrid systems in which other components (e.g. engines, PEM fuel cells, batteries) take care of the dynamic behavior of the demand. In a CTV, loads are highly dynamic with frequent stops. Therefore, SOFC cannot be used in a CTV, at least not as stand-alone application.

**Fig. 4:**
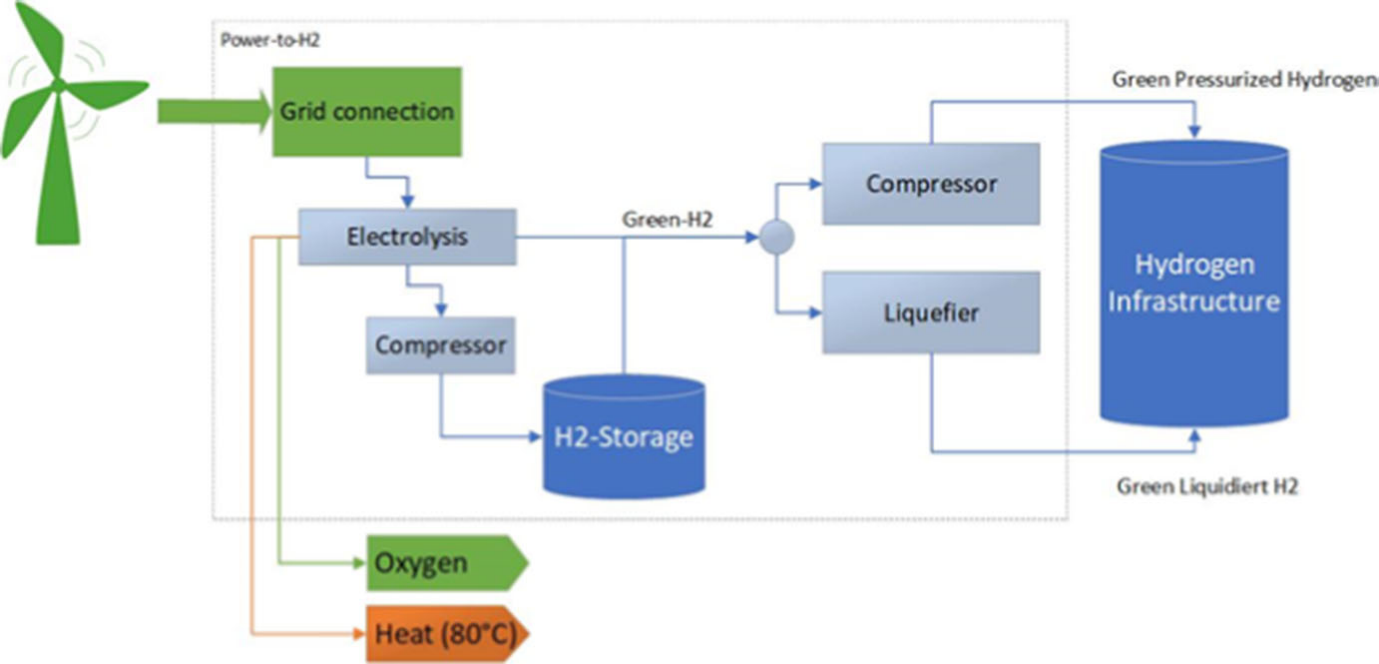
Hydrogen production process based on Bünger et al. (2017)

The premise of this paper is that CTVs would be bunkered by hydrogen produced in OWFs. Figure 4 shows the hydrogen production process based on (Bünger et al. 2017). First, hydrogen is produced through the electrolysis of water. Then, it is transformed into a liquid form both by compressing it and by cooling it in a liquefier. A more thorough description of this process is given in (Fitz et al. 2022). However, in this paper, we only consider the compressed hydrogen, which avoids issue with boil-off related to liquid hydrogen.

## Material

Our work utilizes AIS data, weather data, and vessel characteristics to calculate MDO consumption. We use commercially available AIS data that were collected from terrestrial and satellite sources for the German exclusive economic zone in the North Sea from the year 2020.

Unfortunately, no convention would allow filtering OWF maintenance vessels from AIS data. The AIS data contains data about the ship type, but this information is not standardized. Thus, entries like “Ferry” or “Offshore Supply Vessel” as well as spelling errors are possible. Therefore, we employed following procedure for forming a list of vessels. First, all available data of the 23218 entries, each representing an individual vessel, were filtered based on the vessel dimensions. Then, from the remaining 10327 entries, certain vessel categories were removed, e.g. dredgers. Finally, the remaining 353 entries were manually verified, which resulted in a list of 73 entries. The full list of selected vessels is given in Appendix A. This list is an estimate. An exact number of CTVs to maintain German OWFs could not be determined since this information is not publicly available.

Furthermore, we obtained from a commercial shipping yard a power utilization curve for an exemplary vessel. It represents the percentage of power required to maintain a certain speed, without the effects of weather. We further studied how the weather affects the resistance. For this purpose, we employed the ERA5 weather data-set (Hersbach et al. 2018), which consists of hourly estimation of various weather characteristics. These data are provided by the European Centre for Medium-Range Weather Forecasts (ECMWF).

Section 6 compares the calculated estimates to real MDO usage values. These data were received from a global energy sector company.

## Methodology

The methods for vessel propulsion calculations are well established (MAN 2018). For a vessel to maintain a set speed, it has to produce a sufficient thrust to overcome the resistance. The total resistance *R*_*tot*_ that opposes the vessel’s motion can be calculated with equation (MAN 2018):

1 $$ R_{tot} = R_{calm} + R_{wind} + R_{wave} + R_{x},  $$

where *R*_*calm*_ is the so-called calm water resistance, *R*_*wind*_ wind resistance, and *R*_*wave*_ wave resistance. *R*_*x*_ combines different sources of resistance including those caused by steering, marine fouling, tides, and propeller cavitation.

**Fig. 5:**
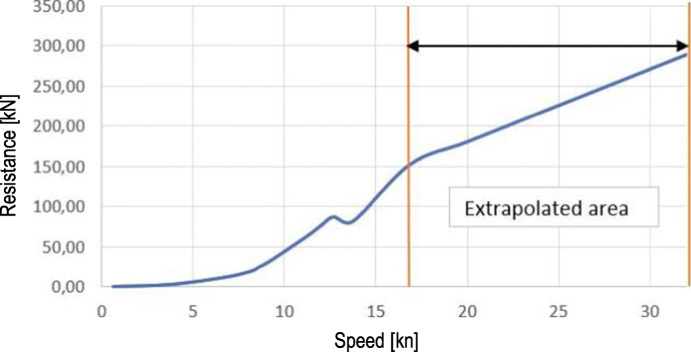
Calm water resistance for a catamaran (Gatin 2021)

**Fig. 6:**
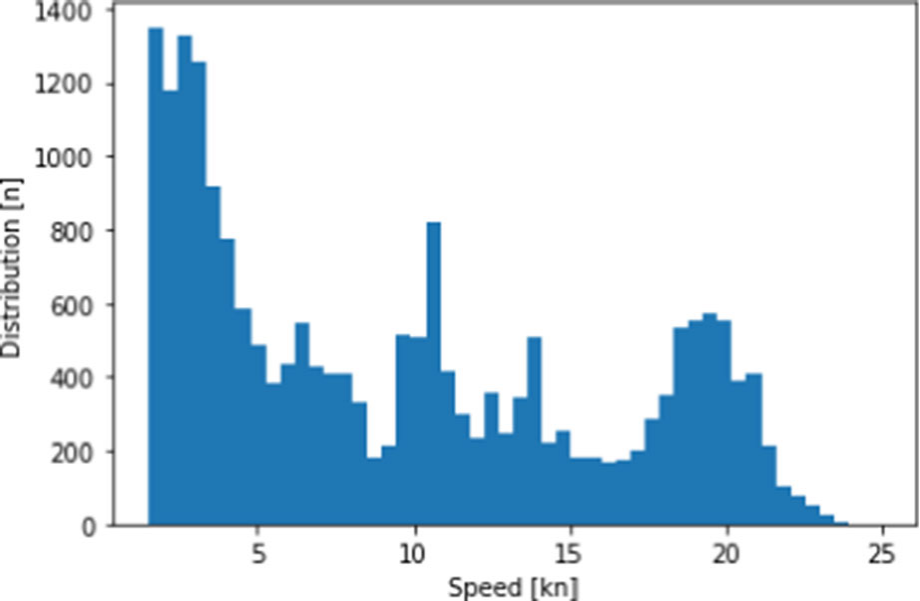
CTV speed distribution in the data set

Calm water resistance is the resistance that a CTV would encounter if the effects of the weather are neglected. An example of a resistance curve for a catamaran is given in Fig. 5. The speeds above 16 nm/h are not available and were extrapolated^2^. This is unfortunate, as Fig. 6 indicates that CTVs in our data cruise with speeds above this limit.

The wind resistance *R*_*wind*_ is calculated with equation (MAN 2018):

2 $$ R_{wind} = \frac{1}{2} \rho_{air} C_{w} A V_{eff}^{2},  $$

where *ρ*_*air*_ is the air density, *A* is the area that is subject to wind resistance, and *V*_*eff*_ is the effective wind speed that takes into account the vessel’s speed (MAN 2018).

Authors are aware of measured *C*_*w*_ values for large ships (ITTC 2014). However, we consider that a semi-sphere is a more representative shape for an aerodynamic CTV than a container or a cruise ship. Therefore, we rely on values taken from (Gasch et al. 2005), which are listed in Table 1.

**Table 1:** Numerical estimates for Eq. 2

Wind direction [^o^]	*C*_*w*_	*A* [m^2^]
0–45	0.33	76
45–90	0.15	111
90–135	− 0.15	111
135–180	− 0.33	76

Area *A* also depends on *V*_*eff*_. We estimated the values based on the dimensions of our reference vessel, also listed in Table 1.

Resistance due to high seas, i.e. weather waves, depends heavily on the ship length and wave length (MAN 2018). In addition, the waves also set the ship in motion. This leads to added resistance as more water is affected by the movement of the ship. Also, more rudder corrections will be needed to stay on course. Due to these reasons, *R*_*wave*_ is currently excluded from the analysis. We further assume that *R*_*x*_ is small compared to other summands in Eq. 1.

The resistance would be transformed into the power demand by calculating the required towing power $P_{T\_i}$ (MAN 2018):

3 $$ P_{T\_i}= R_{tot} V, $$

and estimating the required engine power *P*_*i*_ by taking into account different efficiencies (MAN 2018):

4 $$ P_{i}= \frac{P_{T\_i}}{\eta_{H} * \eta_{S} * \eta_{R} * \eta_{RP}}. $$

The ranges for the efficiency values are as follows: hull efficiency *η*_*H*_ = 0.95 − 1.05, shaft line efficiency *η*_*s*_ = 0.9 − 0.99, rotational efficiency *η*_*R*_ = 1 − 1.07, and free running propeller efficiency *η*_*RP*_ = 0.35 − 0.75 (Łebkowski 2020).

**Fig. 7:**
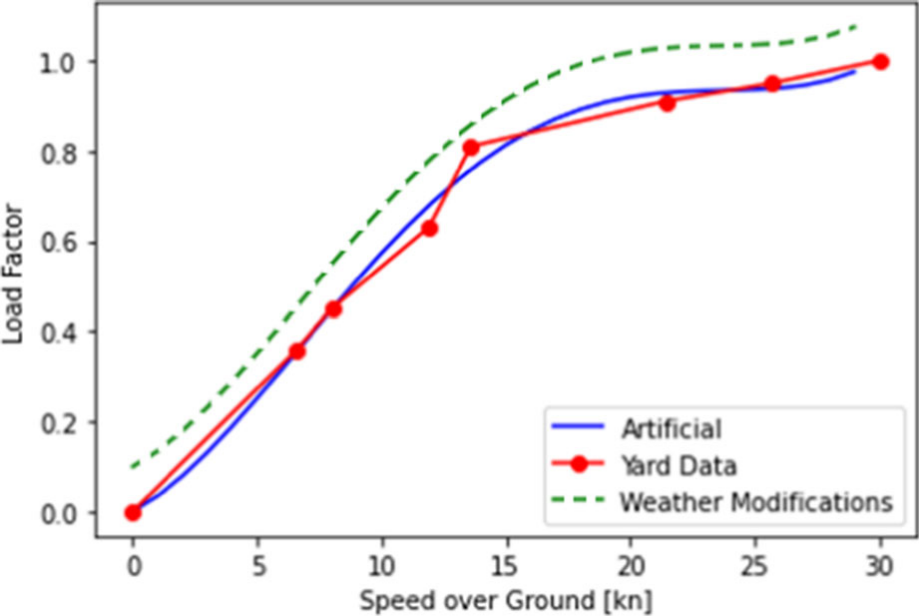
Example of power utilization percentage for different vessel speeds

As mentioned above, there were issues with the available resistance curve, coefficient for Eq. 2, and estimating *R*_*wave*_. Therefore, we relied on a power utilization curve from a commercial shipping yard. In this curve, resistances are already transformed into the power utilization percentage of the engine. In Fig. 7, the red curve shows information from a shipping yard, to which an “artificial” curve is fitted. The newly created plot is based on polynomial interpolation of the yard data. The green curve represents an example of how the weather correction increases the required power to maintain a certain speed.

**Fig. 8:**
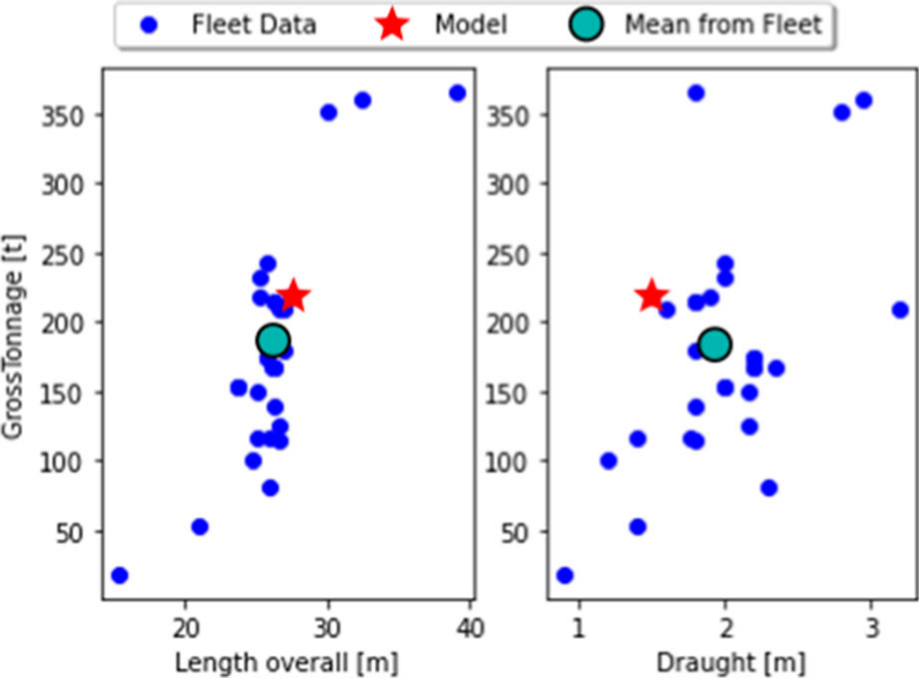
Resistance model vessel parameters compared to fleet data

The utilization curve is for a specific vessel. However, based on its dimensions, the curve provides a good basis for approximating the behavior of other CTVs. Figure 8 depicts the dimensions of this specific vessel compared to the list of vessels we selected for our analysis.

The most important parameters relate to the vessel’s draught and length. Length *L*, width *B*, draught *T*, and volume of displacement Δ are used for calculating a so-called block coefficient *C*_*B*_ (MAN 2018):

5 $$  C_{B} = \frac{\Delta}{L B T}. $$

The authors of (MAN 2018) state that this is the most important coefficient to express the shape of the hull. However, as most CTVs have a multi-hull structure, the width of the vessel is a less important factor than in mono hull vessels. The tonnage of a vessel defines its Δ. As, based on Archimedes’ principle, the weight of an object is equal to the weight of the fluid (water) it displaces.

Power utilization depends also on vessel operations. CTVs consume large amounts of MDO during a so-called ropeless mooring to a wind turbine. In this operation, a CTV pushes against a turbine with 70% power utilization. To consider these operations, we assume that a CTV is pushing when it is stationary (*V* < 1 nm/h) for not more than 30 min. If a CTV is stationary for more than 30 min, we consider it to be in idle mode.

The MDO and hydrogen consumptions are estimated similarly as in Fitz et al. (2022). The mass *m*_*MDO*_ of the consumed MDO is calculated via equation:

6 $$ m_{MDO}= \sum\limits_{i=0}^{n}(P_{ME\_i} * SFOC_{ME\_i} + P_{AE\_i} * SFOC_{AE\_i}) {\Delta} t_{i} , $$

where $P_{ME\_i}$ and $P_{AE\_i}$ are power for the main engine and auxiliary engine during the time step Δ*t*_*i*_. $SFOC_{ME\_i}$ and $SFOC_{AE\_i}$ are instantaneous MDO consumption for these engines during the time step. The mass is calculated by summing the consumption over all time steps 0 - *n*. We made following assumptions:

1. $SFOC_{ME\_i} = 185$ g/kW/h for a modern high-speed vessel (Kristensen 2015);
2. auxiliary power usage results in 150 l MDO consumption per day independent from a CTV use.

Emissions *m*_*e*_ are estimated based on the used amount of MDO with equation:

7 $$  m_{e} = m_{MDO} * f_{e}, $$

where emission factor *f*_*e*_ is specific for each type of emission CO_2_, NO_*X*_, SO_*X*_, and microparticles PM2.5 based on Faber et al. (2021).

The hydrogen consumption *m*_*H*2_ is estimated with equation:

8 $$ m_{H2}= \frac{P_{i} {\Delta} t_{i}}{\eta_{FC} LHV_{H2}} $$

where *P*_*i*_ is the power demand during a time step Δ*t*_*i*_, *η*_*FC*_ = 50*%* is the fuel cell efficiency, and $LHV_{H2} \approx 33.3\frac {\text {kWh}}{\text {kg}}$ is the lower heating value (van Biert et al. 2016).

We assume that the hydrogen is produced by electrolysis, where the process of splitting water molecules between two electrodes utilizes electricity. The required electricity *E*_*EL*_ is calculated with equation

9 $$ E_{EL}= \frac{m_{H2} HHV_{H2}}{\eta_{EL}} , $$

where $HHV_{H2} \approx 39.4\frac {\text {kWh}}{\text {kg}}$ is the higher heating value, and *η*_*FC*_ = 80*%* is the efficiency of a PEM Electrolyser (Shiva Kumar and Himabindu 2019).

The hydrogen is then compressed to storage pressure, which further consumes electricity, but the advantage is lower losses due to boil-off compared to liquid hydrogen. We simplify several possible compression stages of the transportation chain^3^ to one compression stage. In this case, the compression energy *E*_*CP*_ is calculated with equation

10 $$ E_{CP}= m_{H2}*{\eta_{c}}^{-1}*T*\frac{k R_{H2}}{k-1} * ((p_{1} / p_{0})^{\frac{k-1}{k}}-1) , $$

where *η*_*c*_ = 0.8 is the compressor efficiency, *T* = 300 K source temperature, *k* = 1.405 hydrogen specific heat relation, and *R*_*H*2_ = 4.1240 kJ/kg K (Çengel and Boles 2008). Pressures *p*_0_ and *p*_1_ are respectively the output pressure from the electrolyser ≈ 30 ∗ 10^5^ Pa and the pressure in the on-board tank ≈ 650 ∗ 10^5^ Pa.

The total required electricity *E*_*T*_ to produce hydrogen for CTVs is the sum of the electricity consumption. It is given by equation

11 $$ E_{T} = E_{EL} + E_{CP}. $$

## Results

We applied the methods described in Section 4 to the data presented in Section 3 and obtained following results:

- total sailed distance of all vessels 2 ∗ 10^6^ nm (27647 nm per CTV);
- total MDO consumption 40745 t (558 t per CTV);
- total hydrogen consumption 13849 t (187 t per CTV);
- required electricity to produce the hydrogen *E*_*EL*_ = 682.06 GWh, *E*_*CP*_ = 35.66 GWh, thus *E*_*T*_ = 717.72 GWh (9.23 GWh per vessel);
- average CTV utilization time 204 days.

**Fig. 9:**
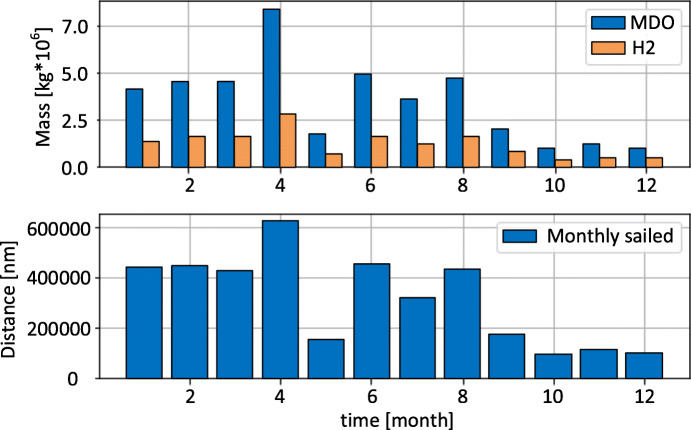
On the top, estimates for MDO and hydrogen consumptions, and on the bottom, vessel activity for individual months in 2020

Figure 9 shows the consumption values for individual months as well as the distance traveled by individual vessels. To put the electricity demand for the hydrogen production in a context, presently the annual electricity production in German offshore wind farms is 26 TWh (WindGuard 2008).

To estimate the effects of the weather, we compared the air resistance and wind resistance. Air resistance *R*_*air*_ only accounts for the calm weather resistance from moving the ship through the atmosphere without any wind (MAN 2018). Wind resistance *R*_*wind*_ is calculated in the same way as *R*_*air*_, but considers the combined speed of the ship and the wind. Both were calculated with Eq. 2 and the ratio:

12 $$ \frac{R_{air}}{R_{wind}} = 0.77. $$

This result means that at least when only the wind speed is considered, the effect of the weather is limited.

**Fig. 10:**
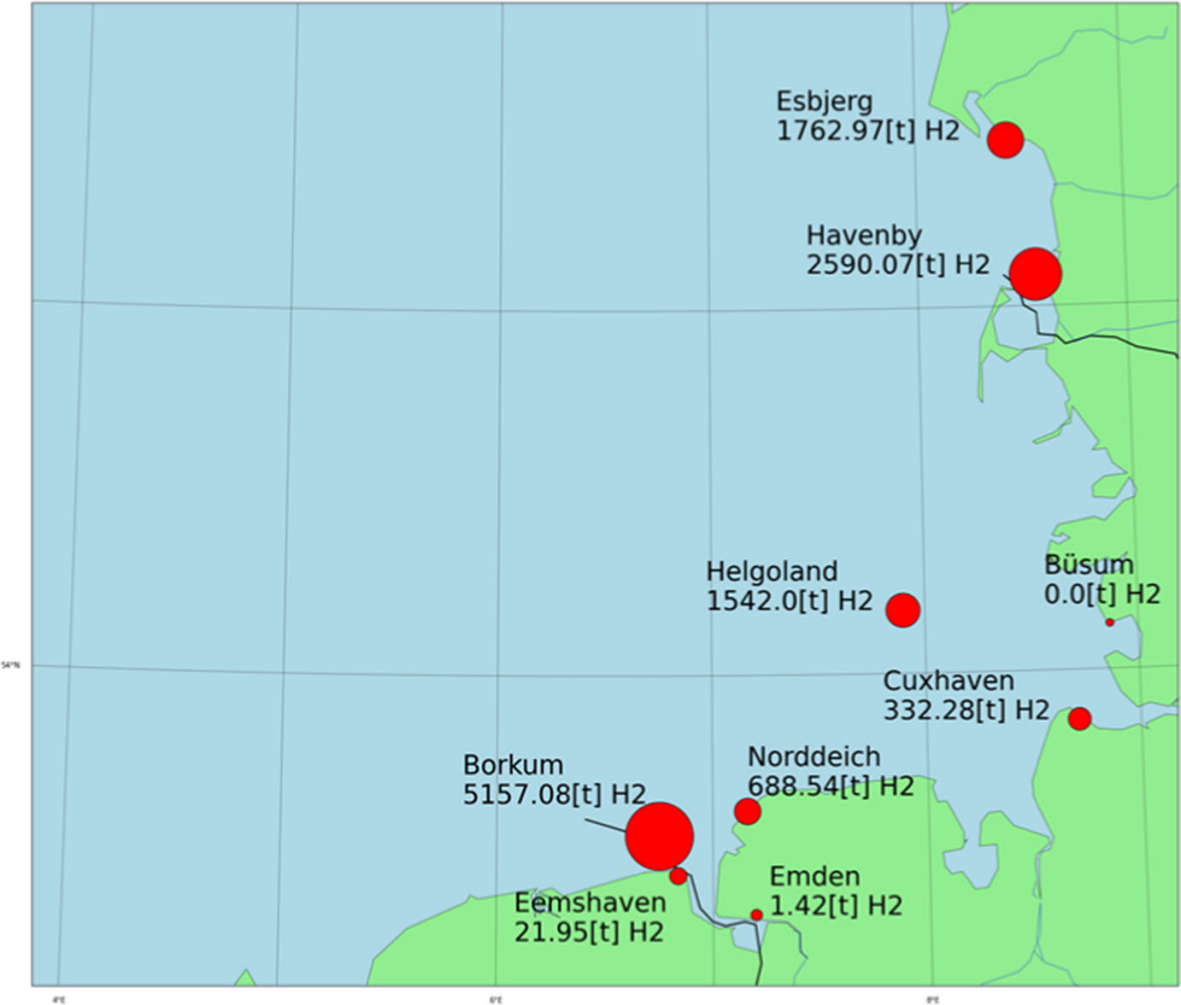
Geographic allocation of the hydrogen demand, based on the assumption that a CTV bunkers all the fuel in the port where it has the most port calls

The hydrogen demand can be geographically mapped based on the assumption where CTVs bunker. We assumed that each vessel bunkers in the port where they have the most port calls. Alternatively, one could allocate the need directly based on the number of calls in different ports. However, we consider this approach as rather unrealistic. The used assumption reflects more closely company practices where vessels have a main port of operations. Figure 10 depicts the hydrogen demand in ports used for maintaining current German OWFs. This kind of estimate will be needed to plan the hydrogen bunkering infrastructure if this means of propulsion becomes the preferred one.

To understand the potential for emission reduction, the total MDO mass was converted to emission estimates using Eq. 7 and the values from (Faber et al. 2021). Following emission values were obtained:

CO_2_: = 126880 t, 1738 t per CTV;
NO_*X*_: = 3732 t, 51 t per CTV;
SO_*X*_: = 57 t, 0.8 t per CTV;
PM2.5: = 92 t, 1.3 t per CTV.

These values are also linked to the economic viability of the propulsion systems due to policies to put a price on emissions.

**Fig. 11:**
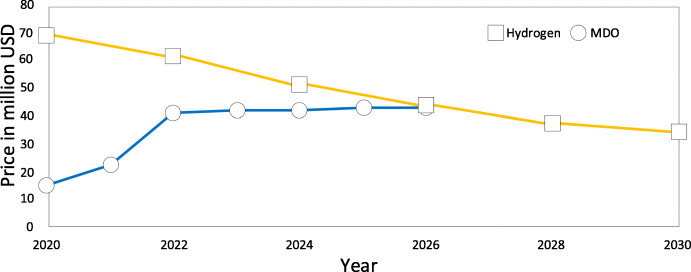
Prediction of hydrogen price based on (DNV 2022) compared to the current marine diesel oil price that includes the German emission certificate costs (BMUV 2019; 2020)

In general, the economic viability of hydrogen-fueled CTV depends on prices of hydrogen and MDO. Figure 11 depicts a prediction of how these prices may change in the future.

The predicted price of hydrogen is taken from (DNV 2022) for renewable hydrogen. The price for hydrogen produced with electricity from offshore wind farms will likely be higher. The reported price has high uncertainties and regional variances. The cost of renewable hydrogen is however estimated to decrease over the time. The reasons for the reduction are that the majority of the current price arises from capital expenditure, which is foreseen to decrease in future. Also, increasing size of turbines in future will increase the annual operating hours, thus leading to higher production rates.

For the price of the marine diesel oil, we used prices from Rotterdam (Ship & Bunker 2022) and predicted that the price stays stable in the future. The only increase in price is related to the German law on emission certificates (BMUV 2019; 2020). There are high uncertainties in these long-term predictions. For example, a report by US Energy Information Administration (EIA 2022) describes several scenarios where oil prices may increase or decrease in the future. However, the reference scenario is that the cost of crude oil will steadily increase over the next 30 years. Even our conservative oil price estimate in Fig. 11 results in a scenario where hydrogen is favorable. Therefore, the margin of difference in favor of hydrogen may be higher.

## Discussion

### Observations on the results

Ideally, the resulting MDO consumption should be compared with the real values to verify the used approach. The authors have nine measurements of annual CTV MDO consumption received from a global energy sector company. However, these data cannot provide statistical proof to verify the method or assess the error. The reasons are that the sample is too small, and it is not randomly chosen, as it consists of vessels operated by a single company. Therefore, the data are only shown as evidence of method viability.

**Fig. 12:**
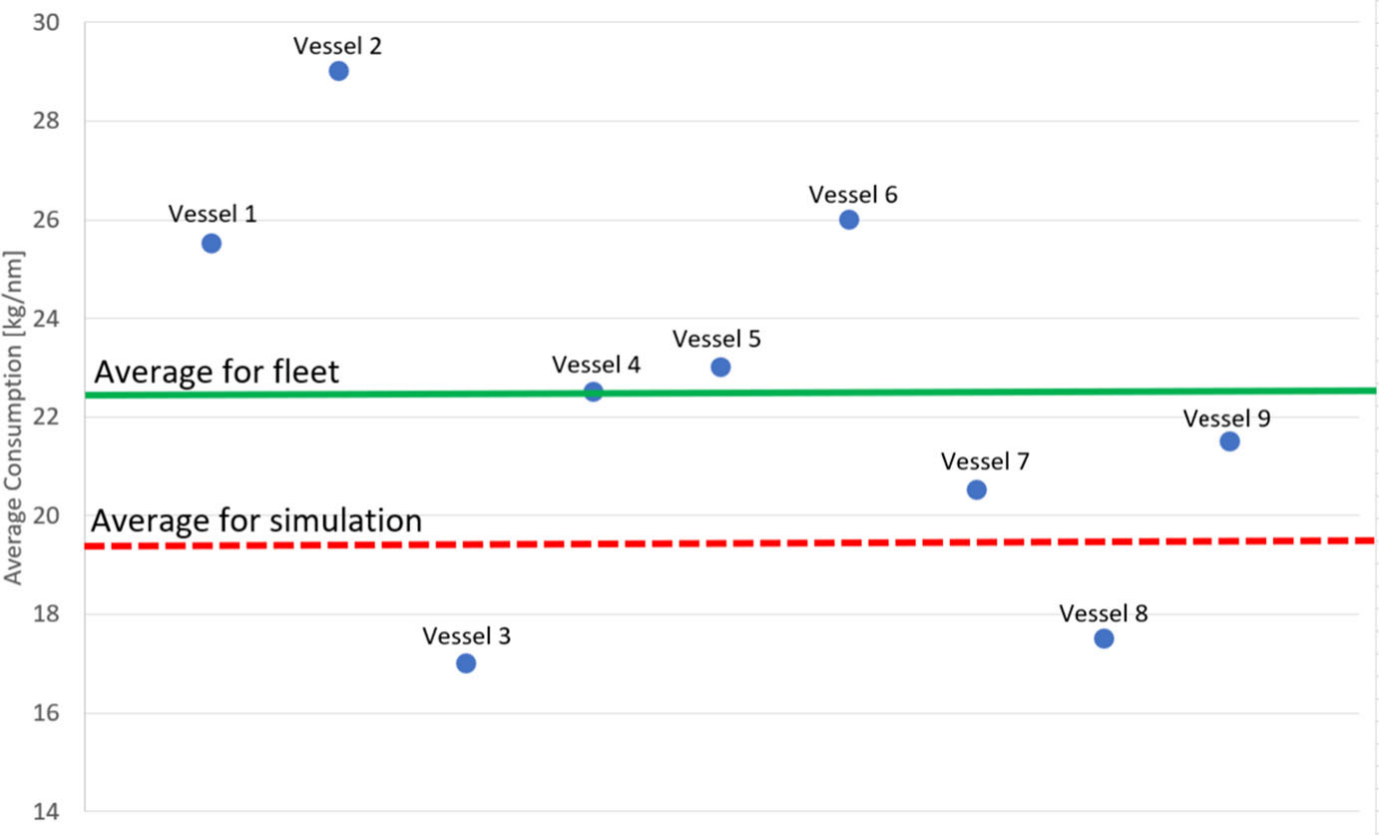
Real MDO consumption data compared to the mean result of the calculation

Figure 12 shows the average MDO consumption calculated from the results in Section 5 compared to actual consumption values from nine measurements from the years 2020 and 2021.

The average calculated consumption is lower than the actual one. This is expected as the resistances caused by weather and maneuvers are excluded from the calculations. However, the difference is lower than what could be assumed based on (Łebkowski and Koznowski 2020) and (Łebkowski 2020).

Interestingly, the results in Fig. 9 further show a dent in activity in the May of 2020. Coronavirus became a global pandemic during the spring of 2020 and caused nations to enact restrictions on movement to slow the spread of infections (Büthe et al. 2020). The authors suspect that the dent is a result of these restrictions.

### Implications of the research

Current decarbonizing efforts call for replacing fossil energy sources including maritime diesel oil with alternatives. This paper studied bunkering CTVs with hydrogen. Section 2 described that hydrogen could be used in small regional vessels and Section 5 showed that hydrogen may be more economical fuel than MDO in the near future.

Realizing these environmental and potential economical benefits depends on abilities to produce and distribute hydrogen. In Germany, the hydrogen strategy (BMWK 2020) and the law on offshore wind energy (Bundesrat 2022) foresee the production of hydrogen in OWFs. However, this does not solve the distribution to CTVs.

There are few alternatives to implement this. Figure 10 assumes that CTVs bunker in the port where they have the most port calls. Other assumptions can be used to modify the geographical distribution of the hydrogen demand. But, the key implication of this research is that the bunkering infrastructure needs to be upgraded to operate hydrogen-fueled CTVs.

## Conclusions and future work

This paper presented a calculation of the amount of hydrogen needed to maintain the current German OWFs and how this demand may be geographically distributed. The estimate was based on a calculation of the MDO consumption of the current fleet, which we further used for estimating the current emissions of these activities. The calculation in turn was based on OWF maintenance operations, as observed in stored Automatic Information System (AIS) data. Based on the predicted price of hydrogen (DNV 2022), bunkering the CTV fleet with hydrogen may become economically advantageous in the future.

The calculation of the current MDO demand was challenging as various hull shapes used in small coastal vessels complicate the estimation of their energy needs. As noted in Section 4, the main current shortcoming is that certain weather effects are excluded from the estimate. Therefore, at the moment the estimated consumption is too small. A comparison with a sample of real data was performed in Section 6. The average consumption for the sample vessels is higher than our estimate. However, the sample is too small and it is not randomly selected. Therefore, statistical methods to measure the error cannot be used.

In this paper, the issue was to form a generic approach that can be applied to all CTVs that are now used in Germany. Future studies may improve the approach. However, it might be more interesting to apply this approach to future scenarios where resistance can be estimated for a selected vessel type rather than all used vessels. This kind of scenario could, for example estimate the needed hydrogen to maintain a selected set of OWFs.

Authors believe that hydrogen should be readily available for OWF maintenance vessels in the future. This is thanks to the German hydrogen strategy that emphasizes the “Power to X concept” (BMWK 2020). Shifting away from MDO to CTVs using hydrogen creates an opportunity to further decrease the carbon footprint of OWFs. Thus, resulting in more environmentally friendly energy production.

## Appendix

### List of vessels in the analysis

This appendix provides the Maritime Mobile Service Identity (MMSI) numbers (ITU 2022) for the vessels included in the MDO consumption assessment of the current CTV fleet to maintain the German OWFs. The numbers are in a comma-separated list: 211471230, 211647900, 211666720, 211755490, 211766470, 211810060, 219014434, 219014436, 219015382, 219016747, 219016873, 219017205, 219018007, 219019933, 219019936, 219020687, 219023785, 219023786, 219024900, 219026749, 219027299, 219142000, 219447000, 219459000, 219463000, 219464000, 219472000, 219513000, 219770000, 219811000, 220626000, 232005600, 232005627, 232006483, 232013230, 232013231, 232013884, 232013885, 232020271, 232020872, 232025959, 232026644, 232027488, 232027489, 232028492, 232031335, 232031394, 235068679, 235080246, 235084702, 235091254, 235095777, 235095778, 235096275, 235098051, 235102028, 235102689, 235103265, 235103385, 235103429, 235108494, 235108711, 235111197, 235111522, 235116577, 235116578, 235117937, 236112545, 244650652, 244830667, 244830668, 253664000, 374011000

## Footnotes

1 The source (Łebkowski and Koznowski 2020) of Fig. 1 is published under the CC-BY-4.0 license.

2 1 nm = 1852 m; 1 nm/h = 1 kn

3 For example, electrolyser – tube-trailer – fueling station – CTV fueling.
